# 3D-printed patient-specific surgical instruments and implants: a technological leap in precision medicine

**DOI:** 10.1097/MS9.0000000000004303

**Published:** 2025-11-24

**Authors:** Muhammad Khizar, Muhammad Zaib, Sharjeel Haider, Hasiba Karimi, Hasibullah Aminpoor

**Affiliations:** a Faculty of Medicine, Georgian American University, Manifest Medical Research Co, Tbilisi, Georgia; b Faculty of Medicine, FMH College of Medicine and Dentistry, Lahore, Pakistan; c Faculty of Medicine, Bezmialem Vakif University, Istanbul, Turkey; d Faculty of Medicine, Kabul University of Medical Sciences “Abu Ali Ibn Sina”, Kabul, Afghanistan

**Keywords:** 3D printing, biocompatible materials, patient-specific implants, precision surgery

## Abstract

Three-dimensional (3D) printing has revolutionized modern surgery by enabling the creation of patient-specific implants and instruments tailored to individual anatomy. Through advanced imaging and computer aided design, 3D-printed devices enhance surgical precision, reduce operative time, and improve functional and aesthetic outcomes across multiple specialties. Clinical evidence from leading countries, including Australia, China, and the United Kingdom, demonstrates successful use of custom titanium spinal and craniofacial implants, restoring complex anatomical structures that standard devices cannot replicate. Technological advances in biocompatible materials such as titanium alloys, PEEK polymers, and bioresorbable composites have expanded the scope of applications, while emerging point-of-care printing facilities allow hospitals to produce customized implants on-site. Despite challenges involving cost, regulatory oversight, and long-term validation, the integration of 3D printing into surgical workflows marks a major step toward personalized medicine. Continued interdisciplinary collaboration and robust clinical trials are essential to establish safety, cost-effectiveness, and broader accessibility of these transformative technologies.

*Dear Editor,*

Innovations in three-dimensional (3D) printing are rapidly transforming surgical practice by enabling the manufacture of custom, patient specific devices. 3D printing (additive manufacturing) can produce implants and instruments matched precisely to a patient’s anatomy^[1]^. This capability is particularly timely as medicine shifts toward personalized care: for example, complex bone tumors, craniofacial defects, or unique joint geometries that defy standard implants are increasingly treated with bespoke 3D-printed solutions. High-resolution imaging (computed tomography/magnetic resonance imaging [CT/MRI]) is used to generate digital 3D models of patient anatomy, which in turn guide the design of implants or cutting guides^[1]^. 3D-printed patient-specific surgical instruments (such as osteotomy or screw-guidance jigs) can improve intraoperative precision by fitting the individual bone surface, reducing reliance on intraoperative estimation. Indeed, systematic reviews have found that 3D-printed patient specific guides and anatomical models significantly reduce operative time and improve accuracy in surgical procedures^[2]^. These benefits arise because the guides conform exactly to the patient’s anatomy, ensuring that planned cuts or drill trajectories are executed as intended.

Clinical applications of patient-specific implants span many specialties. In spine surgery, for example, surgeons have used custom 3D-printed titanium vertebral bodies to reconstruct extensive bone loss. Australian case series report successful reconstruction of C2 vertebrae with patient-specific 3D-printed implants for malignancy-associated spinal collapse^[3]^. Similarly, in China, a series of patients undergoing total en bloc spondylectomy for spinal tumors received 3D-printed titanium vertebral prostheses. Early results showed restored spinal stability with few implant failures^[4]^. These examples demonstrate how advanced printing enables solutions in scenarios (e.g., multi-level bone tumor resections) that were previously inoperable or required radical sacrifice of anatomy. In cranio-maxillofacial surgery, patient-specific titanium plates and implants are already routine. A UK multicentered survey found that most surgeons perceived 3D-printed patient-specific mandibular or orbital implants as superior to conventional plates, noting improved surgical accuracy, shorter operating time, and better aesthetic outcomes^[5]^. No procedure studied in that survey was rated worse with printed implants. These reports from major centers in Australia, China, the UK, and beyond highlight the global momentum of patient-specific 3D-printed devices.

Technically, patient-specific implants are fabricated from advanced biomaterials under strict design controls. Metal implants are often made of biocompatible titanium alloys (e.g., Ti6Al-4V) via powder-bed fusion (laser or electron beam) methods, while high-performance polymers such as PEEK are used for loadbearing applications like custom cranial plates. 3D printing allows complex porous geometries: for instance, latticed structures with optimized porosity can match the elastic modulus of bone and enhance osteointegration^[6]^. Indeed, recent reviews note that custom 3D-printed spine fusion cages can be tailored in geometry and porosity to optimize bone ingrowth and mechanical fit^[6]^. Biodegradable materials (polylactic acid or tricalcium-phosphate composites) are also under active investigation for resorbable plates or scaffolds in craniofacial reconstruction. Some hospitals have established point-of-care 3D-printing facilities: one recent report describes in-hospital fabrication of polymeric patient-specific craniofacial implants, enabling same-site production and faster turnaround^[7]^. Regulatory pathways are evolving to accommodate these devices. For example, the US FDA recognizes 3D-printed implants and guides within its 510(k)-clearance process. In April 2024, the FDA cleared the first 3D-printed patient-specific PEEK cranial implant, underscoring the maturation of this technology. However, point-of-care printing also raises considerations about quality control, sterilization, and biocompatibility, so multidisciplinary collaboration with engineers and compliance with medical-device regulations is essential.

From a clinical perspective, patient-specific 3D-printed tools and implants offer clear advantages. Surgeons report that customized cutting guides and models improve preoperative planning and intraoperative confidence, often shortening procedures^[2,5]^. Custom implants precisely fill irregular bone defects, which can reduce stress on adjacent joints or soft tissues and potentially lower revision rates. For patients with severe deformities or tumors, these implants restore anatomy that generic devices could not, yielding improved functional and neurologic outcomes. On the other hand, barriers remain: manufacturing costs, the need for specialized software and hardware, and limited high-level evidence beyond case series. Early adopters emphasize that well-designed prospective studies are still needed to quantify long-term outcomes and cost-effectiveness.

In summary, 3D printing of patient-specific surgical instruments and implants represents a major advance in surgical technology. It is already improving patient care by enabling bespoke solutions to complex anatomical problems, with demonstrated benefits in accuracy and operative efficiency^[2,5]^. Future directions should include rigorous trials of clinical impact, development of next-generation biomaterials (e.g., bioactive ceramics, resorbable metals), and integration of bio-printed tissues. As this field grows, surgical teams must stay informed and engage with engineers to realize the full potential of personalized, 3D-printed surgical devices. We confirm that no AI tools were utilized in the conduct or writing of this manuscript, in accordance with the TITAN checklist.^[8]^

## Data Availability

Not applicable for this review.

## References

[1] U.S. Food and Drug Administration. Printing of Medical Devices. 2023. Accessed 30 September 2025. Available from: https://www.fda.gov/medicaldevices/products-and-medicalprocedures/3d-printing-medical-devices

[2] Diment LE Thompson MS Bergmann JH. Clinical efficacy and effectiveness of 3D printing: a systematic review. BMJ Open 2017;7:e016891. 10.1136/bmjopen-2017-016891 PMC5778284 29273650

[3] Hunn SAM Koefman AJ Hunn AWM. 3D-printed titanium prosthetic reconstruction of the C2 vertebra: techniques and outcomes of three consecutive cases. Spine (Phila Pa 1976) 2020;45:667–72. 31809469 10.1097/BRS.0000000000003360

[4] Wang X Sun S Jiang Y. Early clinical efficacy of 3D-printed artificial vertebral body in spinal reconstruction after total en bloc spondylectomy for spinal tumors. BMC Musculoskelet Disord 2024;25:926. 39558232 10.1186/s12891-024-08069-7 PMC11575014

[5] Goodson AMC Parmar S Ganesh S. Printed titanium implants in UK craniomaxillofacial surgery. Part II: perceived performance (outcomes, logistics, and costs). Br J Oral Maxillofac Surg 2021;59:320–28. 33280945 10.1016/j.bjoms.2020.08.088

[6] Lewandrowski KU Vira S Elfar JC. Advancements in custom 3Dprinted titanium interbody spinal fusion cages and their relevance in personalized spine care. J Pers Med 2024;14:809. 39202002 10.3390/jpm14080809 PMC11355268

[7] Maintz M Tourbier C de Wild M. Patient-specific implants made of 3D printed bioresorbable polymers at the point-of-care: material, technology, and scope of surgical application. 3D Print Med 2024;10:13. 38639834 10.1186/s41205-024-00207-0 PMC11031859

[8] Agha RA Mathew G Rashid R. TITAN Group. Transparency in The reporting of Artificial INtelligence – the TITAN guideline. Prem Sci 2025;10:100082.

